# Local and Loco-Regional Anesthesia in Patients Who Underwent Thyroid and Parathyroid Surgery

**DOI:** 10.3390/jcm14051520

**Published:** 2025-02-24

**Authors:** Marco Fiore, Gianluigi Cosenza, Domenico Parmeggiani, Francesco Coppolino, Fausto Ferraro, Maria Caterina Pace

**Affiliations:** 1 Department of Women, Child and General and Specialized Surgery, University of Campania “Luigi Vanvitelli”, Via Luigi De Crecchio, 2, 80138 Naples, Italy; gianluigi.cosenza@studenti.unicampania.it (G.C.); francesco.coppolino@unicampania.it (F.C.); fausto.ferraro@unicampania.it (F.F.); mariacaterina.pace@unicampania.it (M.C.P.); 2 Department of Translational Medical Sciences, University of Campania “Luigi Vanvitelli”, Via Leonardo Bianchi, 80131 Naples, Italy; domenico.parmeggiani@unicampania.it

**Keywords:** thyroid surgery, parathyroid surgery, regional anesthesia, cervical epidural anesthesia, cervical plexus block, regional anesthesia

## Abstract

**Background/Objectives**: Globally, thyroid and parathyroid diseases are common and often require surgery. This review evaluates the current literature on the use of regional anesthesia in these surgeries, highlighting its advantages, limitations, and areas requiring further research. **Methods**: MEDLINE (via PubMed) and ResearchGate, the largest academic social networks, were utilized to retrieve literature on the topic. **Results**: Fifteen studies with few patients and largely uncontrolled on the use of loco-regional anesthesia (LRA) not combined with general anesthesia (GA) were found. Meanwhile, twenty-two better quality studies involving several patients on LRA combined GA were found. **Conclusions**: LRA, in combination with GA, has been proven to be the most reliable evidence for reducing opioid use and postoperative nausea and vomiting. LRA, not combined with GA, has been used in a few well-conducted studies; it seems to be feasible to use even in patients with severe systemic disease. Future controlled studies will need to validate its effectiveness and safety.

## 1. Introduction

Thyroid and parathyroid hormones are fundamental in human metabolism. Their role in metabolic pathways, growth, development, cognition, energy homeostasis, and temperature regulation is well known [1]. In large population-based studies, disorders in their functions are in the range of 2–6%. Globally, thyroid and parathyroid dysfunctions are common endocrinological and oncological issues [2].

The incidence of thyroid nodules in the general population is 30–50%. The median age of these patients is 40–60 years, with a gender distribution of 3–4:1 (female: male) [3].

Goiters are common as well. When they become voluminous, apart from being a cosmetical problem, they may cause compression of the respiratory and upper digestive tracts and the recurrent laryngeal nerve (RLN). Surgery is frequently indicated in these types of pathologies, especially when the goiters become intrathoracic, causing compression of important structures, such as vessels and trachea.

From 1990 to 2019, age-standardized prevalence rates indicated a global increase in thyroid cancer (TC), with around 18.3 million cases in 2019 [4].

Female preponderance is seen in parathyroid dysfunctions as well. Primary hyperparathyroidism is most common in post-menopausal women, while in hypoparathyroidism, most of the patients (>75%) are the result of surgery for thyroid disorders, which are more common in women. The average age of patients with primary hyperparathyroidism is about a decade older than that of hypoparathyroidism patients [5].

Surgeries of these glands are one of the most performed. Every year, in European countries, between 45,000 and 60,000 thyroidectomies are performed.

These surgeries are traditionally performed under general anesthesia (GA). However, increasing attention has been given to local anesthesia (LA) and loco-regional anesthesia (LRA) techniques. The advantages of these techniques are to avoid the risks associated with general anesthesia, minimize postoperative pain, and enhance recovery. These techniques are particularly intriguing for individuals who are at high surgical risk (ASA III or IV) or intolerant to general anesthesia. According to the principle of the last guidelines for preoperative assessment of adults undergoing elective noncardiac surgery, it is suggested that every patient undergoes a personalized anesthesiologic evaluation [6].

The most common regional anesthesia techniques for thyroid and parathyroid surgeries include superficial and deep cervical plexus blocks, as well as local anesthetic infiltration. The scope of this study is to describe the actual literature regarding LA and LRA in thyroid and parathyroid surgery in combination or not with GA, reporting the advantages and limitations of the included studies and analyzing the areas requiring further research. The description of the anesthetic techniques (e.g., cervical block) goes beyond the scope of this study.

## 2. Materials and Methods

A search of the biomedical literature was conducted to review the published clinical data on regional anesthesia in patients who underwent thyroid and parathyroid surgery. In primis, MEDLINE (via PubMed) was searched, with no temporal limits, for articles using the following terms “(local OR regional OR locoregional) AND (anesthesia) AND ((thyroid) OR (parathyroid)) AND surgery”. Additionally, ResearchGate, which has the highest number of active users in academic social networks, was utilized to find manuscripts that were not included in MEDLINE. Eligible studies were studies without a control group or with a control group (both randomized trials and non-randomized), published in full in peer-reviewed journals and English language with no restrictions on their setting or publication date. We included all the studies identified, but we did not evaluate their quality or use any other systematic literature review methodology. For each study, we identified the year of publication, the clinical setting, the study methodology (type), the total number of participants (patients enrolled), the participants assigned to LA/LRA, the group of participants assigned to a control (the control arm), the outcome of the various studies and their time of exploration, and the results and any adverse events reported.

## 3. Results

### 3.1. Local/Loco-Regional Anesthesia Not Combined to General Anesthesia

For more than three decades, local anesthesia has been explored in thyroid surgeries (Table 1). In 1991, Hochman et al. performed 43 sequential thyroidectomies: 21 were performed using LA and 22 under GA. For the authors, LA is a valid option for reducing patients’ in-stay in less invasive procedures [7]. Successively, Lo Gerfo et al. performed 236 bilateral neck explorations (BNEs) from 1988 to 1999 under LA. Intravenous sedation was used to give more comfort to the patient. This study demonstrated that LA is a safe alternative to GA in performing BNE in patients with thyroid disease and nonlocalized adenoma [8].

Ishiguro et al. had, in their case series, which were performed between 1997 and 2002, 18 parathyroidectomies under LA. They demonstrated that LA can be a valid strategy in performing minimally invasive parathyroidectomies in a day surgery regimen, improving patients’ quality of life [9].

Spanknebel et al., in a large prospective study on 1025 patients, studied the efficacy of LA on thyroidectomy. The types of interventions went from total thyroidectomies to lobectomies and partial resections. As in previous studies, a key to the success of LA in performing thyroid surgery is in the experience of the surgeon. In this study, the authors established that LA is safe and applicable in almost all patients, including those with a high ASA score [10].

Snyder et al. divided 58 patients into two groups: 29 performed thyroidectomy under GA and 29 under LA plus monitored anesthesia care (MAC). The main objective was to study the effectiveness and safety of LA for this type of procedure. Secondary outcomes were patients’ satisfaction and cost benefits of LA. There were no conversions from LA to GA, and post-anesthesia care unit (PACU) stay was less in the LA group. Cost savings were 315 USD per patient receiving LA plus MAC. Patients’ satisfaction was similar between the two groups [11].

Banasiewicz et al. performed 37 subtotal bilateral thyroidectomies and 12 lobectomies or partial lobectomies using LA with 1% lignocaine. In their setting, LA was a valid alternative to GA due to technical issues. This anesthesia was well tolerated by the patients, especially when intravenous sedatives were combined with LA. The authors mention some technical difficulties when patients had stimulus for coughing, making surgery more difficult [12].

Kim et al. divided 60 patients into two groups: 30 patients received LA plus MAC and 30 patients GA. The main outcomes explored were postoperative nausea and vomiting (PONV), postoperative discomfort, postoperative pain, odynophagia, dyspnea, and patient satisfaction. There were no differences between the groups in postoperative pain, odynophagia, dyspnea, and patient satisfaction levels. The LA plus MAC group had fewer PONV episodes, throat discomfort, and voice changes [13].

Haugen et al., in their case series, performed 28 thyroidectomies in LA. An important factor that influenced the efficacy of the procedure was the surgeons’ experience in LA. An intraoperative pain score between 0 and 10 was evaluated, with a median score of 3.4. The success rate of LA was 96%; only one patient required GA due to airway issues. Seventy-one percent of the patients tolerated surgery with only LA and did not require sedation at all. The amount of lidocaine 2% used was 24 mL [14].

Mamede et al., in their randomized controlled trial (RCT) conducted in 2006, evaluated the efficacy of superficial cervical plexus block (SCPB), cost-effectiveness, laryngotracheal injuries, and patient satisfaction in patients undergoing hemithyroidectomy. The main results of this study were that differences in hospital stay between the two groups were not statistically significant (*p* < 0.05). The mean duration of anesthesia was higher in the SCPB group. Costs were lower in the LA group. In the SCPB group, there were no laryngotracheal injuries; in the GA group, 51% of patients presented them [15].

Lombardi et al., in a case series of five patients who underwent video-assisted thyroidectomy after signing informed consent, had surgery performed under LRA instead of GA. After adequate premedication, using diazepam 0.2 mg/kg taken orally 45 min before surgery, a cervical block was performed using bupivacaine 0.25% and carbocaine 0.5%. The technique used was a landmark technique, injecting a total of 20 mL. Five mL of LA was injected by the surgeon to infiltrate the upper pole and the thyroid capsule. At the end of surgery, ketorolac 30 mg and ranitidine 100 mg were administered. No conversion to general anesthesia occurred. The visual analog scale (VAS) was used to assess pain. Thirty minutes after the beginning of the operation, the median VAS score was 2.0, and at the end of the procedure, the mean score was 1.5. No complications were observed, and only one patient required ketorolac as a rescue analgesic [16].

Stephen et al. performed bilateral superficial cervical plexus block (BSCPB) with 1.0% Xylocaine with adrenaline (2–3 mL/kg body weight) in thyroid surgery in rural hospitals. In this setting, LA demonstrated to be a valid alternative to GA, in terms of safety, efficacy, and spending review [17].

In a prospective trial conducted in 2009, Inabnet et al.performed 10 thyroidectomies under LA. The main outcomes were the evaluation of the feasibility of the procedure under LA and the evaluation of the efficacy of the external branch of superior laryngeal nerve (EBSLN) monitoring and voice handicap index-10 (VHI-10) score for 3 weeks after surgery. The monitoring of EBSLN under LA helped the surgeon to identify the nerve and avoid injuries in 70% of the procedures. With the isolation and avoidance of ESBLN, patients did not experience changes in their voice after 3 weeks, as evaluated by the VHI-10 score [18].

Suri et al. studied 95 patients undergoing thyroid and parathyroid surgeries. They divided these patients into two groups: 64 received standard GA and 31 received BSCPB+ sedation. Patients who underwent surgery with the LRA technique had advantages in the recovery including a faster return to normal daily activity and satisfaction with the anesthesiologic management [19].

Rahman et al. performed nine cases of surgery for thyroid disorders under superficial or deep cervical plexus block. The advantages observed were less intraoperative bleeding. The avoidance of endotracheal intubation was a time-saving procedure, a faster recovery, and capacity for oral nutrition. All of the patients studied in this case series were operated under regional anesthesia combined with intravenous sedation [20].

Santosh et al. conducted 29 thyroid surgeries only under regional anesthesia. In 20 patients, deep cervical plexus block (DCPB) was performed, and in 9, cervical epidural anesthesia (CEA) was the anesthesiologic option. Patients were comfortable during the procedure, and no episodes of PONV were reported after surgery. Between the two techniques, there were no statistical differences in the time of surgery and patients’ satisfaction. The surgeon was also able to monitor the vocal cords’ status [21].

**Table 1:** Loco-regional anesthetics (LRA) not combined with general anesthesia (GA).

Author	Year	Setting	Study Type	N. Patients Enrolled	LR Technique	Control Arm	Outcome Explorated	Evaluation Time	Results	Adverse Events
Hochman et al. [7]	1991	University Hospital	Retrospective	43	LA (21)	GA (22)	Reducing recovery time	NS	LA enhance discharge time	Not reported
Lo Gerfo et al. [8]	1999	University Hospital	Case series	236	LA+ sedation (236)	/	Patient compliance	0, 3, 6 h	Acceptable	Not reported
Ishiguro et al. [9]	2002	University Hospital	Case series	18	LA (18)	/	Patient compliance	NS	Acceptable	GA (1)
Spanknebel et al. [10]	2005	University Hospital	Prospective	1686	LA (330)	GA (1356)	Efficacy, safety and LOS	NS	Not inferior	RLN injuries (30), hematoma (5), HC (1), tracheostomy (1), SSI (1)
Snyder et al. [11]	2006	University Hospital	RCT	58	LA + MAC (29)	GA (29)	Efficacy, safety and LOS	NS	Not inferior	RLN injury in each group (1)
Banasiewicz et al. [12]	2011	University Hospital	Case series	49	LA (49)	/	Efficacy	NS	Acceptable	Not reported
Kim et al. [13]	2017	University Hospital	RCT	60	LA + MAC (30)	GA (30)	PONV, safety, postoperative pain	NS	Not inferior	Not reported
Haugen et al. [14]	2019	Medical Center	Case series	28	LA (27)	/	Patient compliance, intraoperative pain	NS	Acceptable	Airway complication (1)
Mamede et al. [15]	2006	University Hospital	RCT	42	SCPB (21)	GA (21)	Efficacy, safety, anesthesia duration, patient compliance	NS	Not inferior LOS. Anesthesia duration higher in LR group. Costs were lower in the LR group.	Not reported
Lombardi et al. [16]	2003	University Hospital	Case series	5	BSCPB (5)	/	Intra- and postoperative VAS score; postoperative non-steroideal anti-inflammatory drug (NSAID) consumption	0, 6, 18, 24 h	VAS score had a median value of 2 in the intraoperative and 1.5 at the end of the procedure	Not reported
Stephen et al. [17]	2008	University Hospital	Case series	11	BSCPB (11)	/	Efficacy	NS	Not inferior	Not reported
Inabnet et al. [18]	2009	University Hospital	Case series	10	BSCPB + DCPB (10)	/	Efficacy	3 weeks	Acceptable	Not reported
Suri et al. [19]	2010	University Hospital	RCT	95	BSCPB + sedation (31)	Standard GA (64)	Recovery advantages	NS	LR experienced a return to work sooner and normal energy levels.	Not reported
Rahman et al. [20]	2011	University Hospital	Case series	9	BSCPB or DCPB (9)	/	Bleeding, operative time, recovery advantages	/	Acceptable	Temporary dysphagia (1)
Santosh et al. [21]	2015	University Hospital	RCT	29	DCPB (20)	Cervical epidural anesthesia (CEA) (9)	Efficacy	NS	Not inferior	Not reported

### 3.2. Local/Loco-Regional Anesthesia Combined with General Anesthesia

Dieudonne et al. performed an RCT on 90 patients undergoing elective thyroid surgery. In the group block, BSCPB was performed at the end of surgery. The main outcomes were the evaluation of pain scores, numeric rating scale 11 (NRS-11), during PACU admission, and the total dose and request of opioids, such as morphine. Median values of NRS were lower in patients receiving LA. The bupivacaine group had a smaller proportion of patients receiving opioids (66.0% vs. 90.0%). The authors concluded that BSCPB could relieve postoperative pain but not provide optimal analgesia alone [22].

Andrieu et al. evaluated the efficacy of bilateral SCPB executed before thyroid surgery in GA. Three groups were randomized to receive saline, ropivacaine 0.487% or ropivacaine 0.478% plus clonidine 5 mcg ml. Sufentanil was given if vital parameters provided a suspicion of pain, and all patients received paracetamol for 24 h after surgery. Pain score was evaluated every 4 h after surgery (NRS) and nefopam was used as rescue therapy if NRS > 4. This study showed that in the group receiving SCPB with ropivacaine, with or without clonidine, the use of opioids in the intraoperative phase (*p* < 0.0001), and rescue therapy in the postoperative (*p* = 0.03) was significantly reduced [23].

Kale et al. evaluated the efficacy of BSCPB when executed before or after surgery. Patients were divided into three groups receiving BSCPB pre- or post-surgery, or no block at all. All patients were induced in GA. Patients receiving LA had a lower postoperative VAS score, with a mean value of 2.27–2.66. Fentanyl requirements were lower in the group that had the block executed before surgery (103 ± 0.8 mcg). The group that received BSCPB in the postoperative phase was the one that had a later request for analgesics, and both groups receiving LA had less incidence of PONV [24].

Liu et al., in their randomized controlled trial, evaluated the efficacy and safety of opioid-free anesthesia (OFA) combined with BSCPB, confronting it with opioid-based anesthesia in thyroid surgery. In both groups, GA was performed, and in the OFA group, BSCPB was executed after intubation. The primary outcome was the incidence of nausea, which occurred in two patients in the OFA group and thirteen patients in the control group. Vomiting did not occur in the OFA group but occurred in five patients in the control arm. VAS score was lower in the OFA group when the patients were in PACU and 2 h and 4 h after surgery, but no significant difference was observed 24 h after surgery (*p* = 1.000). In the ward, one patient in the OFA group received analgesics, and eight patients in the opioid group had a rescue analgesic. The QoR-40 score, a recovery questionnaire, was higher in the OFA group. No adverse events related to LA were observed [25].

Suh et al. compared the efficacy between BSCPB and combined (superficial and deep) cervical plexus block administered before thyroid surgery. Patients in ASA I and II risk were divided into a control Group CO, a BSCPB Group S, and a combined C group. In the S group, 18 mL of 0.25% bupivacaine was administered, and in the C group, 14 mL was administered in the BSCPB and 4 mL in the deep cervical plexus block (DCPB). The average concentration of remifentanil was significantly reduced in the S group as well as incision pain at rest and swallowing measured 0, 2, and 4 h after surgery (*p* < 0.05). The requirement of opioids as analgesics in the recovery room was significantly reduced in Group S compared with Groups C and CO (*p* < 0.05). The incidence of PONV was reduced in Group S, and patients’ satisfaction was higher in this group [26].

In a prospective cohort study, Woldegerima et al. evaluated the efficacy of analgesic BSCPB for thyroid surgery performed under GA. The block was performed just before induction, and 10 mL of 0.25% bupivacaine was injected. Median NRS-11 scores were reduced in patients receiving LRA, and the time for the first analgesic request was more prolonged in these patients (132.3 min vs. 71.4 min). PONV occurred in 27% of the patients in the block group and 35.1% of the patients in the non-block group, but no statistical difference was observed [27].

Shih et al. randomly assigned patients who were candidates for elective thyroid operations to receive BSCPB with isotonic saline (Group A), bupivacaine 0.5% (Group B), or levobupivacaine 0.5% (Group C) after induction of general anesthesia. The main outcomes evaluated were intraoperative anesthetics, the consumption of postoperative rescue analgesia and VAS score, and the incidence of PONV, hospital stay, and discomfort in swallowing. Patients in Group A received higher doses of desfluorane (5.8% vs. 3.9% vs. 3.8%) in Groups A, B, and C, respectively. In patients receiving LA, it took longer to receive adjunctive analgesics. VAS score was lower in Groups B and C. Hospital stay was lower in Groups B and C [28].

Gurkan et al. evaluated postoperative opioid consumption and median VAS score of 50 patients undergoing thyroid surgery who were randomly assigned to a group receiving BSCPB or standard GA. Opioid-related side effects, such as PONV, were examined as well. Morphine consumption at 6, 12, and 24 h postoperatively was higher in the control group. VAS scores for pain were similar in both groups. Six patients in the SCPB group had nausea, and four of them had vomiting as well. In the control group, eight patients had nausea and four had vomiting. Seven patients in the SCPB group had hoarseness following the block [29].

In a randomized controlled trial, Yao et al. evaluated the effects of BSCPB on the quality of recovery through the QoR-15 questionnaire. Secondary outcomes studied were acute postoperative pain, time to first rescue analgesia, number of patients requiring rescue analgesia, length of post-anesthesia care unit (PACU) stay, incidence of PONV and dizziness, and patients’ satisfaction. The global postoperative quality of recovery questionnaire (QoR-15) scores in the SCPB group were higher (median difference 8%). VAS scores in the 24 h postoperative were lower in the patients receiving regional anesthesia, especially in the first 8 h, but no statistical differences were found 24 h after surgery. The median time for the first rescue analgesia was longer in the SCPB group than in the control group (18.8 h vs. 8.1 h), and postoperative morphine use was reduced. Preoperative block performance reduced PACU stay and improved patients’ satisfaction [30].

Steffen et al. studied the impact of BSCPB executed before or after surgery on postoperative pain, analgesic use, and length of hospital stay. Patients receiving BSCPB had less pain than the placebo group, not depending on the timing of the block. There was no difference between the groups on analgesic use, and the length of hospital stay was the same between the block group and placebo [31].

Ozgun et al. divided 60 patients into Group 1, who received thyroid surgery under a standard GA, and Group 2, where the patients received a BSCPB with levobupivacaine 0.5%. Patients in the LA group at 2, 6, 12, and 24 h postoperatively had lower NRS scores, required less rescue analgesia, and had a lower consumption of tramadol. Two cases of postoperative subcutaneous emphysema were reported in Group 2, which regressed spontaneously after 12 h. No other complications related to BSCPB were reported [32].

Karakis et al. randomized 46 patients undergoing total thyroidectomy in a group receiving GA and another one receiving GA + BSCPB with bupivacaine 0.25%. The outcomes evaluated were the intraoperative remifentanil requirements and VAS score post extubation, at 15 min, 30 min, and 1, 2, 6, 12, 24, and 48 h. Total tramadol, paracetamol, and ondansetron use were reported as well. The intraoperative opioid consumption was significantly lower in the LA group (*p* = 0.009), as well as the postoperative pain scores (*p* < 0.01). Postoperative opioid and analgesic requirements were lower in the BSCPB group, as well as the incidence of PONV [33].

Goulart et al. studied the ability of BSCPB to control pain and reduce the side effects of GA in patients undergoing thyroidectomy. In this RCT, 100 patients were divided into Group 1, receiving GA alone, and Group 2, receiving GA plus BSCPB. Hemodynamic parameters were better controlled in Group 2 during PACU stay at 15, 30, 45, and 60 min. These patients had better pain control and lower opioid consumption. The incidence of nausea and vomiting was lower in patients receiving BSCPB [34].

Eti et al. divided 45 patients into three groups. In Group I, after the induction of general anesthesia, BSCPB with 0.25% bupivacaine was performed; in Group II, wound infiltration with LA was performed; and in Group III, no regional block was administered. In this study, there were no differences in VAS scores among the different groups, and the total patient-controlled analgesia (PCA) meperidine was no different. The first analgesic request time was longer in Group with I. The incidence of PONV was similar between the three groups [35].

Herbland et al. divided 111 patients into three groups of 37. In Group CONT, no block was performed; in Group PRE, BSCPB was performed before surgery under GA; and in Group POST, BSCPB was performed after surgery. The main outcomes evaluated by the authors were total morphine administration and consumption in the first 36 h and pain intensity scores. No statistical differences resulted in NRS scores between the different groups, as well as the first analgesic requirement in the PACU and morphine requirements [36].

Karthikeyan et al. divided 60 patients undergoing thyroid surgery into a control group (Group S) and two other groups. One of these (Group B) received BSCPB with only bupivacaine 0.25%, and the other group (Group BC) received 0.25% bupivacaine plus clonidine 1 mcg/kg. The main outcomes were Intra- and postoperative analgesic requirements, postoperative pain scores, incidence of PONV, and sedation. BSCPB, performed with only LA or with adjuvants, was effective in reducing both Intra- and postoperative analgesic requirements, and clonidine reduced the incidence of PONV. The median VAS score between the three groups was the lowest in Group BC. The difference in time for the first analgesic requirement was statistically different (*p* = 0.002) between Group S and Group B and between Group S and Group BC [37].

Kesisoglou et al. randomized 100 patients undergoing total thyroidectomy into two groups: Group S did not receive LA, and in Group R, BSCPB was performed under GA with 15 mL of 0,75% of plain ropivacaine. A reduction in median VAS score was noted at all timings, except 12 h in Group R. Additional analgesia (dextropropoxyphene hydrochloride) was required for seven patients in Group S and eight patients in Group R. Sufentanil was required in two patients in Group S and one patient in group R [38].

Sardar et al. performed BSCPB with 15 mL of 0.25% bupivacaine for each side in patients undergoing thyroid surgery. When confronted with the control group, BSCPB did not reduce the median VAS score for the first 24 h, nor intravenous analgesic doses. Patients who received LA had fewer episodes of PONV, and the first analgesic time requirement was longer. The authors concluded that BSCPB did not decrease analgesic requirements after thyroid surgery [39].

Cai et al. randomized 135 patients undergoing thyroid surgery in a control group, receiving a standard GA, and a group receiving BSCPB with 20 mL of ropivacaine 0.5%. The main outcomes explored were the incidence of PONV, the request for rescue antiemetics, and postoperative VAS scores, evaluated every 4 h after surgery for the first 24 h. The incidence of PONV was significantly lower in the ropivacaine group (*p* = 0.001), as well as the request of antiemetics (*p* = 0.015). VAS scores were lower in the ropivacaine group at 0, 4, and 8 h (*p* < 0.05), but no differences were observed at 12, 16, and 24 h after surgery [40].

Negmi et al. evaluated the effect of BSCPB in patients undergoing total thyroidectomy. The main outcomes evaluated were postoperative pain, patient satisfaction, and the total amount of morphine administered. The measures were repeated every 4 h for the first 24 h after surgery. When confronted with the control group, patients receiving LA had lower median VAS scores and a reduced amount of morphine required to control postoperative pain, and the patients were more satisfied with the analgesia [41].

Ahiskalioglu et al. randomized 60 patients undergoing thyroidectomy into three groups. Group C (control) received a standard GA, Group SC received a BSCPB with bupivacaine 0.25% and Group SC + T received oral tizanidine and BSCPB. This study showed that BSCPB was effective in reducing postoperative pain scores, opioid consumption, and side effects. Patients who received tizanidine had reduced early postoperative opioid consumption, posterior neck pain, and occipital headache [42].

Aweke et al. studied the efficacy of BSCPB in 66 patients undergoing thyroid surgery. The main outcomes evaluated were postoperative NRS scores, time to the first analgesic requirement, and the incidence of PONV. Patients in the LA group showed reduced postoperative pain scores, a statistically significant difference in the time of first analgesic requirement (*p* = 0.001), and a reduced incidence of PONV [43].

The main findings from studies focusing on LRA combined with GA are summarized in Table 2.

## 4. Discussion

The purpose of this review is to report on current concepts regarding regional anesthesia’s role in thyroid and parathyroid surgery. On the other hand, in regional anesthesia, the surgeon may encounter difficulties during surgery due to the lack of muscle release caused by the absence of neuromuscular blocking agents. Surgical difficulties may increase the risk of complications such as bleeding and possible reoperations. The limitation of this review is that it does not reveal any controlled studies on the topic and the BSCPB was described in only one case series as effective in reducing bleeding, saving time, allowing faster oral nutrition, and early mobility [20].

Another concern is that in other surgical settings, the discharge of loco-regional patients may be delayed further by the additional need for sedation; the length of stay and direct costs increase significantly when the boarding period in post-anesthesia care units is prolonged [44,45]. Interestingly, in thyroid surgery even the mean duration of anesthesia is higher in the SCPB group than the GA group; there are no statistical differences in hospital stay between the two groups [15]. Additionally, patients who were in the LR group experienced a faster return to work [19]. The rapid return to work may have an economic advantage, but unfortunately, this has not been explored. The cost and the training requirements for successfully implementing LA and LRA have not been investigated in the retrieved studies, which are other important aspects.

Future studies on the economic impact of different techniques are needed.

In the last two decades, intraoperative neural monitoring (IONM) has become a standard method for monitoring the RLN during thyroid surgery [46]. Unfortunately, there are no studies of IONM in patients who undergo thyroid surgery under LRA not combined with GA.

The most reliable evidence is the use of LRA in combination with GA; it appears that the use of LRA combined with GA has an opioid-sparing impact, enhancing recovery and reducing PONV [25,26,47].

The analgesic efficacy in the perioperative period after thyroid surgery and the reduced opioid use and PONV is confirmed by a recent systematic review with meta-analysis, including 2273 patients enrolled in 31 studies published until January 2022, comparing the effect of BSCPB to no block or placebo block with saline [47].

The CDC recognized the need for guidelines on pain management to improve appropriate opioid prescribing while minimizing opioid-related risks, as the United States is experiencing an opioid epidemic [48]. However, the use of regional loco anesthesia seems to be desirable to reduce the prescription of opioid analgesics.

The outcomes of previous systematic reviews on the topic were focused mainly on opioid use, analgesia efficacy, and PONV control [47,49]. Complications (the most reducible ones being recurrent nerve injury and bleeding), postoperative difficulties, and reoperations were not analyzed. Furthermore, only studies that used BSCPB as an RA technique were included [47,49]. In our review, we included all the techniques described in the literature. From our narrative review, both scoping and systematic reviews comparing local and loco-regional anesthesia in thyroid and parathyroid surgery can provide clearer guidance for clinical practice. Due to the heterogeneous outcomes of the retrieved studies, conducting a meta-analysis may be more challenging.

## 5. Conclusions

This LRA, in combination with GA, has been proven to be the most reliable evidence for reducing opioid use and postoperative nausea and vomiting in thyroid and parathyroid surgeries.

LRA, not combined with GA, has been used in a few well-conducted studies. Although it seems feasible to use it even in patients with severe systemic disease, future well-conducted controlled studies are needed to validate its effectiveness and safety.

## Figures and Tables

**Table 2:** Loco-regional anesthetics (LRA) combined with general anesthesia (GA).

Author	Year	Setting	Study Type	N. Patients Enrolled	LR Technique	Controlled Arm	Outcome Explorated	Evaluation Time	Results	Adverse Events
Dieudonne et al. [22]	2001	University Hospital	RCT	90	BSCPB (47)	Standard GA (40)	Postoperative pain; opioid consummation.	0, 2, 6, 24 h	Reduced postoperative pain; opioid consummation in LRA	Not reported
Andrieu et al. [23]	2007	University Hospital	RCT	87	BSCPB (58)	Standard GA (29)	Intra/Postoperative pain; opioid and nefopam consumption; PONV	0, 3, 6, 9, 12, 18, 24 h	Reduced intra/postoperative pain; opioid and nefopam consumption in LRA	PONV LRA (23); PONV GA (8); RLN injury LRA (10), RLA injurie GA (4).
Kale et al. [24]	2015	Teaching Hospital	RCT	60	BSCPB pre- (20) or post-surgery (20)	Standard GA (20)	Postoperative pain; opioid consumption; PONV	0, 1, 2, 4, 8, 12, 18, 24, 36, 48 h	Reduced postoperative pain; PONV and opioid consumption in LRA	PONV LRA (1); PONV GA (6).
Liu et al. [25]	2023	Municipal Hospital	RCT	75	BSCPB and OFA anesthesia (33)	Opioid-based anesthesia (33)	Safety; postoperative pain; PONV	2, 4, 6, 24 h	Postoperative pain and PONV reduced in LRA	PONV LRA (2); PONV GA (18)
Suh et al. [26]	2009	University Hospital	RCT	90	BSCPB (30) and combined SCPB and DCPB (30)	Standard GA (30)	Postoperative pain; opioid consumption; PONV	0, 4, 6, 12, 24 h	Reduced postoperative pain and opioid consumption in LRA	PONV LRA (13); PONV GA (14)
Woldergerima et al. [27]	2020	University Hospital	Prospective	74	BSCPB (37)	Standard GA (37)	Postoperative pain; opioid consumption	0, 2, 6, 12, 24 h	Reduced postoperative pain and opioid consumption in LRA	PONV LRA (10); PONV GA (13)
Shih et al. [28]	2010	Tertiary Care Hospital	RCT	162	BSCPB (106)	Standard GA + placebo (56)	Desfluorane consumption; postoperative pain and rescue analgesia; LOS.	2, 6, 10, 14, 18, 22, 26 h	Reduced desfluorane compsuntion; postoperative pain and rescue analgesia; LOS in LRA	Transient diaphragmatic paresis LRA (1); PONV LRA (34/106); PONV GA (21/56)
Gurkan et al. [29]	2014	University Hospital	RCT	50	BSCPB (25)	Standard GA (24)	Postoperative pain; opioid consumption	1, 6, 12, 24 h	Reduced postoperative pain; opioid consumption in LRA	Transient hoarseness LRA (7) and ear lobe numbness LRA (1)
Yao et al. [30]	2019	University Hospital	RCT	74	BSCPB (37)	Standard GA (37)	Postoperative pain; recovery quality and rescue analgesia; LOS	0.5, 1, 2, 4, 8, 24 h	Reduced postoperative pain; opioid consumption and LOS with a better recovery quality in LRA	PONV LRA (1); PONV GA (17)
Steffen et al. [31]	2010	Teaching Hospital	RCT	183	BSCPB (93)	Standard GA (90)	Postoperative pain and analgesic consumption; LOS	Q8h for 3 days	Postoperative pain and analgesic consumption in LRA; no differences in LOS	Transient ear lobe numbness LRA (41).
Ozgun et al. [32]	2022	State Hospital	RCT	60	BSCPB (30)	Standard GA (30)	Postoperative pain and rescue analgesia; opioid consumption	2, 6, 12, 24 h	Reduced postoperative pain and rescue analgesia, opioid consummation in LRA	Subcutaneous emphysema LRA (2).
Karakis et al. [33]	2019	University Hospital	RCT	46	BSCPB (23)	Standard GA (23)	Postoperative pain and opioid, paracetamol, and ondansetron consummation	0.25, 0.5, 1, 2, 6, 12, 24, 48 h	Reduced postoperative pain and opioid paracetamol, and ondansetron consummation in LRA	PONV LRA (2); PONV GA (11)
Goulart et al. [34]	2019	University Hospital	RCT	100	BSCPB (50)	Standard GA (50)	Intra/Postoperative pain; opioid consumption. PONV	0.25, 0.5, 0.75, 1, 4, 8, 12-h	Reduced intra/postoperative pain; opioid consumption and PONV in LRA	PONV LRA (5); PONV GA (22)
Eti et al. [35]	2006	University Hospital	RCT	45	BSCPB (15)	Standard GA ± LA (30)	Opioid consumption	1, 2, 4, 8, 12, 16, 20, 24 h	No differences in opioid consumption.	PONV LRA (7); PONV GA (10)
Herbland et al. [36]	2006	University Hospital	RCT	111	BSCPB (74)	Standard GA (37)	Postoperative pain; opioid consumption. PONV	q4h for 1.5 days	No differences in postoperative pain and opioid consumption	Transient left brachial paresthesia (1) and arm partial motor block (1) in LRA. PONV LRA (22/74); PONV GA (15/37)
Karthikeyan et al. [37]	2012	University Hospital	RCT	60	BSCPB (40)	Standard GA (20)	Intra/Postoperative pain; opioid consumption. PONV	2, 4, 6, 8, 16, 24 h	Reduced intra/postoperative pain; opioid consumption and PONV in LRA	PONV LRA (11/40); PONV GA (10/20)
Kesisoglou et al. [38]	2009	University Hospital	RCT	100	BSCPB (50)	Standard GA (50)	Postoperative pain; opioid consumption.	0, 3, 6, 9, 12, 24 h	Reduced postoperative pain and opioid consumption in LRA (first 12 h)	Not reported
Sardar et al. [39]	2013	University Hospital	RCT	60	BSCPB (30)	Standard GA (30)	Postoperative pain; opioid consumption.	/	No differences in postoperative pain an opioid consumption.	Not reported
Cai et al. [40]	2012	University Hospital	RCT	135	BSCPB (68)	Standard GA (67)	Postoperative pain; opioid consumption. PONV	0, 4, 8, 16, 24 h	Reduced postoperative, opioid consumption and PONV in LRA	Transient Horner syndrome (12) in LRA; PONV LRA (29); PONV GA (51)
Negmi et al. [41]	2005	Teaching Hospital	RCT	50	BSCPB (25)	Standard GA (25)	Postoperative pain; opioid consumption; patient satisfaction.	0, 4, 8, 16, 24 h	Reduced postoperative pain, opioid consumption with excellent patient satisfaction in LRA	Not reported
Ahiskalioglu et al. [42]	2018	University Hospital	RCT	60	BSCPB (40)	Standard GA (20)	Postoperative pain; opioid consumption; safety	0, 1, 2, 4, 8, 12, 24 h	Reduced postoperative pain; opioid consumption in LRA	Transient hoarseness LRA (2/40); PONV LRA (14/40); PONV GA (7/20)
Aweke et al. [43]	2018	University Hospital	RCT	66	BSCPB (33)	Standard GA (33)	Intra/Postoperative pain; opioid consumption; PONV	3, 6, 12, 24 h	Reduced intra/postoperative pain, opioid consumption and PONV in LRA	PONV LRA (20); PONV GA (25)

## Data Availability

The corresponding author can provide databases and literature screening upon valid request.
